# Metal-organic-frameworks derived cobalt embedded in various carbon structures as bifunctional electrocatalysts for oxygen reduction and evolution reactions

**DOI:** 10.1038/s41598-017-05636-y

**Published:** 2017-07-13

**Authors:** Binling Chen, Guiping Ma, Yanqiu Zhu, Yongde Xia

**Affiliations:** 1 College of Engineering, Mathematics and Physical Sciences, University of Exeter, Exeter, EX4 4QF United Kingdom; 2 State Key Laboratory of Chemical Resource Engineering, Beijing Laboratory of Biomedical Materials, Beijing University of Chemical Technology, Beijing, 100029 P.R. China

## Abstract

A series of nanocomposites of cobalt embedded in N-doped nanoporous carbons, carbon nanotubes or hollow carbon onions have been synthesized by a one-step carbonization of metal-organic-framework ZIF-67. The effect of the carbonization temperature on the structural evolution of the resulting nanocomposites has been investigated in detail. Among the as-synthesized materials, the cobalt/nanoporous N-doped carbon composites have demonstrated excellent electrocatalytic activities and durability towards oxygen reduction reaction in alkaline medium. Compared to the benchmark Pt/C catalyst, the optimized Co@C-800 (carbonized at 800 °C) exhibited high oxygen reduction reaction activity with an onset potential of 0.92 V, and a half-wave potential of 0.82 V. Moreover, the optimized Co@C-800 also showed enhanced electrocatalytic activity towards oxygen evolution reaction from water splitting, with a low onset potential of 1.43 V and a potential of 1.61 V at 10 mA cm^−2^ current density. This work offered a simple solution to develop metal-organic-framework-derived materials for highly efficient electrochemical applications.

## Introduction

Developing highly efficient electrocatalysts for energy technologies has attracted increasingly intense attention due to the worldwide continuously growing demand in energy^1^. Electrocatalytic oxygen reduction reaction (ORR) and oxygen evolution reaction (OER) play key roles in the next generation of energy conversion and storage applications, such as fuel cells^2–5^, metal-air batteries^6–8^ and water splitting^9–12^. To date, the most efficient catalyst for ORR and OER usually contains high-cost and scarce precious metals^13^. Therefore, it is vital and compelling to explore and develop highly efficient and cost-effective electrocatalysts towards both ORR and OER. Consequently, noble-metal-free catalysts such as non-precious transition metal compounds and heteroatom-doped carbons have been studied^14, 15^. For example, Dai *et al*. reported mesoporous carbon foam codoped with phosphorus and nitrogen as an excellent bifunctional electrocatalyst for ORR and OER^14^. Wang *et al*. prepared cobalt-embedded nitrogen-doped carbon/nanodiamond electrocatalysts for high performance ORR and OER in alkaline media^15^.

Metal organic frameworks (MOFs) have attracted attention because of their tuneable structures, versatile functionalities and fascinating textural properties^16–23^. Recently, inspired by their molecular-like organic-inorganic crystal structure, MOFs are used as self-sacrificed templates for synthesising metal nanoparticles embedded within the heteroatom-doped (typically N-doped) nanoporous carbon matrices through thermal decomposition. This strategy is advantageous due to the homogeneous dispersion of metal nanoparticles within the heteroatom-doped porous carbon matrix and the ease of synthesis directly from the MOFs without any additional precursors. So far, various metal/heteroatom-doped carbon composites synthesized from the MOFs at relatively low temperatures below 1100 °C have been reported in literature^24–37^, and their applications for ORR have also been studied^24, 27, 28, 30–36^. For instance, cobalt/porous carbon composites derived from ZIF-67 exhibited excellent ORR performance^24, 27, 28, 37^. However, the research on the MOF derivatives as bifunctional ORR/OER catalysts has been rarely reported. In addition, the synthesis of MOF derivatives at relatively high temperatures (above 1000 °C) has not been explored, considering the complicated interactions between the formed metal particles and the carbon species and the structure change of carbons during the carbonization process^29, 38^.

In this work, we report the structural evolution of MOF-derived materials ranging from cobalt-embedded in N-doped porous carbons to hollow carbon onions. ZIF-67 was applied as the precursor in this work, taking advantage of its high porosity, high nitrogen content, and rich content of cobalt ions. The structure of the as-synthesized materials was modulated by the carbonization temperatures. In addition, both the ORR and OER performances of these MOF-derived materials have been systematically evaluated. Among the synthesized materials, the cobalt-embedded porous N-doped carbon composite (Co@C-800) exhibited excellent ORR and OER catalytic performance due to its favourable porous nanostructure, N-doping effect and homogeneous cobalt dispersion.

## Experiment

### Materials synthesis

ZIF-67 was synthesized from cobalt nitrate hexahydrate and 2-methylimidazole in water, following a modified procedure^39^. The composites were prepared by temperature programmed carbonization of ZIF-67 in Ar atmosphere. Typically, 0.25 g ZIF-67 was heated to 600–2000 °C at a heating rate of 10 °C min^−1^ under Ar atmosphere with a flow rate of 100 ml/min in a tube furnace, then kept at the target temperature for 3 h, and followed by cooling down to room temperature. The composites were labelled as Co@C-T, where T means the carbonization temperature.

### Materials characterization

X-ray diffraction (XRD) was measured under a Cu Kα radiation (40 kV-40 mA) with a step size of 0.02° and a step time of 1 s. N_2_ gas sorptions curves were measured by a Quantachrome Autosorb-iQ gas sorptometer. The powdered samples were firstly evacuated under vacuum at 200 °C for 3 h. N_2_ sorption analysis was then carried out at −196 °C. Brunauer-Emmett-Teller (BET) method was used to calculate the specific surface area. The Raman spectra were measured by using a 532 nm laser excitation with 6 mW laser power. X-ray photoelectron spectroscopy (XPS) was applied using a Kratos AXIS ULTRA spectrometer. Scanning electron microscopy (SEM) was measured by a Philips XL-30 machine at a voltage of 20 kV. Transmission electron microscopy (TEM) was measured by JOEL-2100 at a voltage of 200 kV.

### Electrochemical measurements

The electrocatalytic performance was measured by cyclic voltammograms (CV), linear sweep voltammograms (LSV) and chronoamperometry tests by a potentiostat CHI 760D machine. The CHI 760 D machine is coupled with a rotating disk electrode (RDE) system. All the tests were measured in a three-electrode electrochemical cell, where the counter electrode is a platinum wire, the reference electrode is an Ag/AgCl/KCl, and the working electrode is a ϕ3 mm glassy carbon electrode (GCE) covered with studied materials. The GCE can be obtained by adding a 5 μL of the catalyst ink. The ink was obtained by ultra-sonication of 1 mg of the catalyst into 0.5 mL 0.05 wt% in alcohol Nafion solution. The measurement was carried in O_2_/N_2_ saturated 0.1 M KOH solution. The reported electrode potential is relative to the reversible hydrogen electrode (RHE) potential, which can be converted by the equation: E_RHE_ = E_(Ag/AgCl)_ + 0.9646 V^40^.

Koutecky-Levich plots were analysed at different potentials. The number of electrons transferred (n) can be obtained from the slopes of their linear-fit lines based on the K-L equations^41, 42^.

## Results and Discussion

### Structural characterization

**Figure 1:**
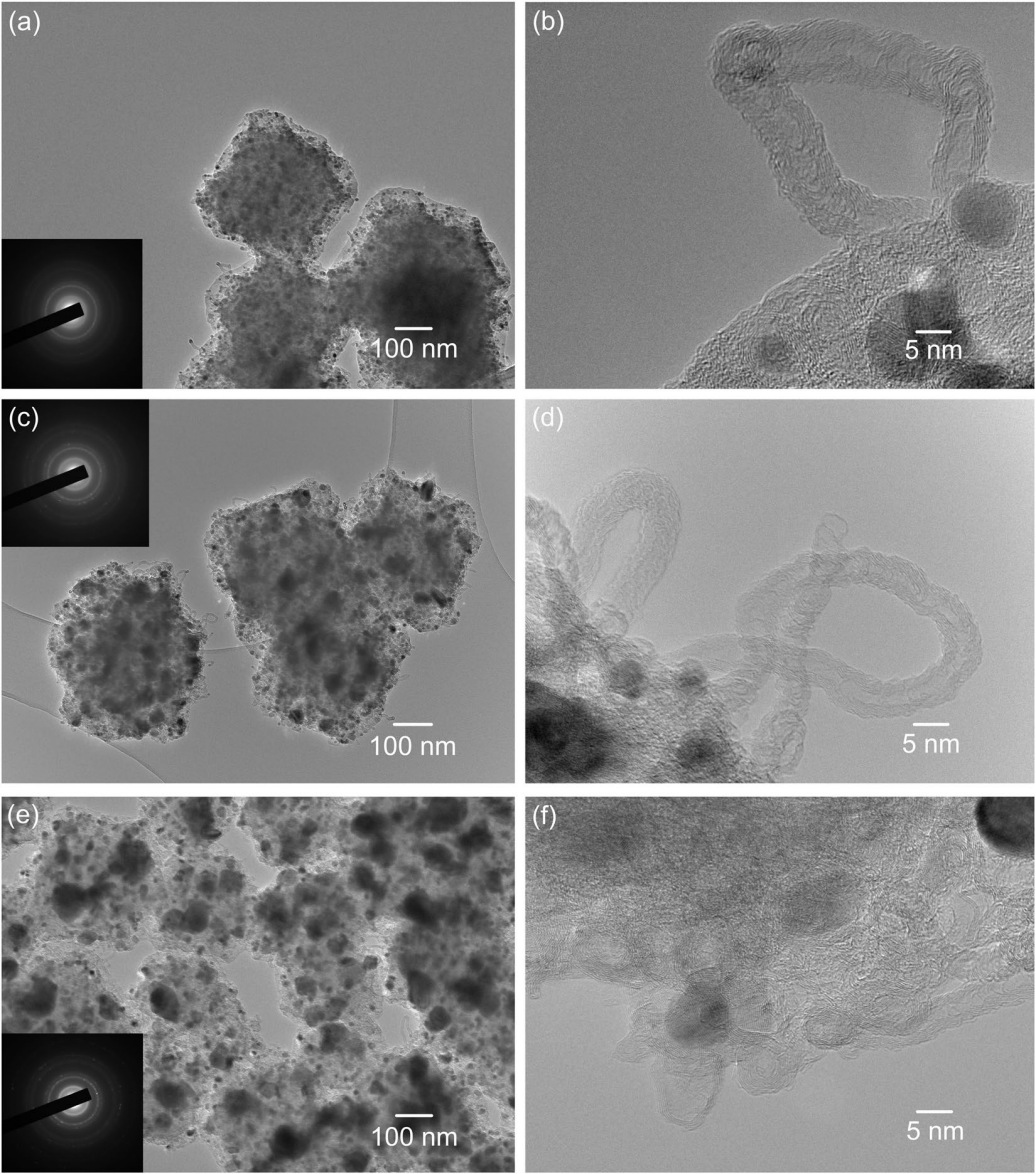
TEM images of the samples generated under relatively low carbonization temperatures: (**a,b**) Co@C-600; (**c,d**) Co@C-800 and (**e,f**) Co@C-1000. Inset: the SAED patterns for each sample.

**Figure 2:**
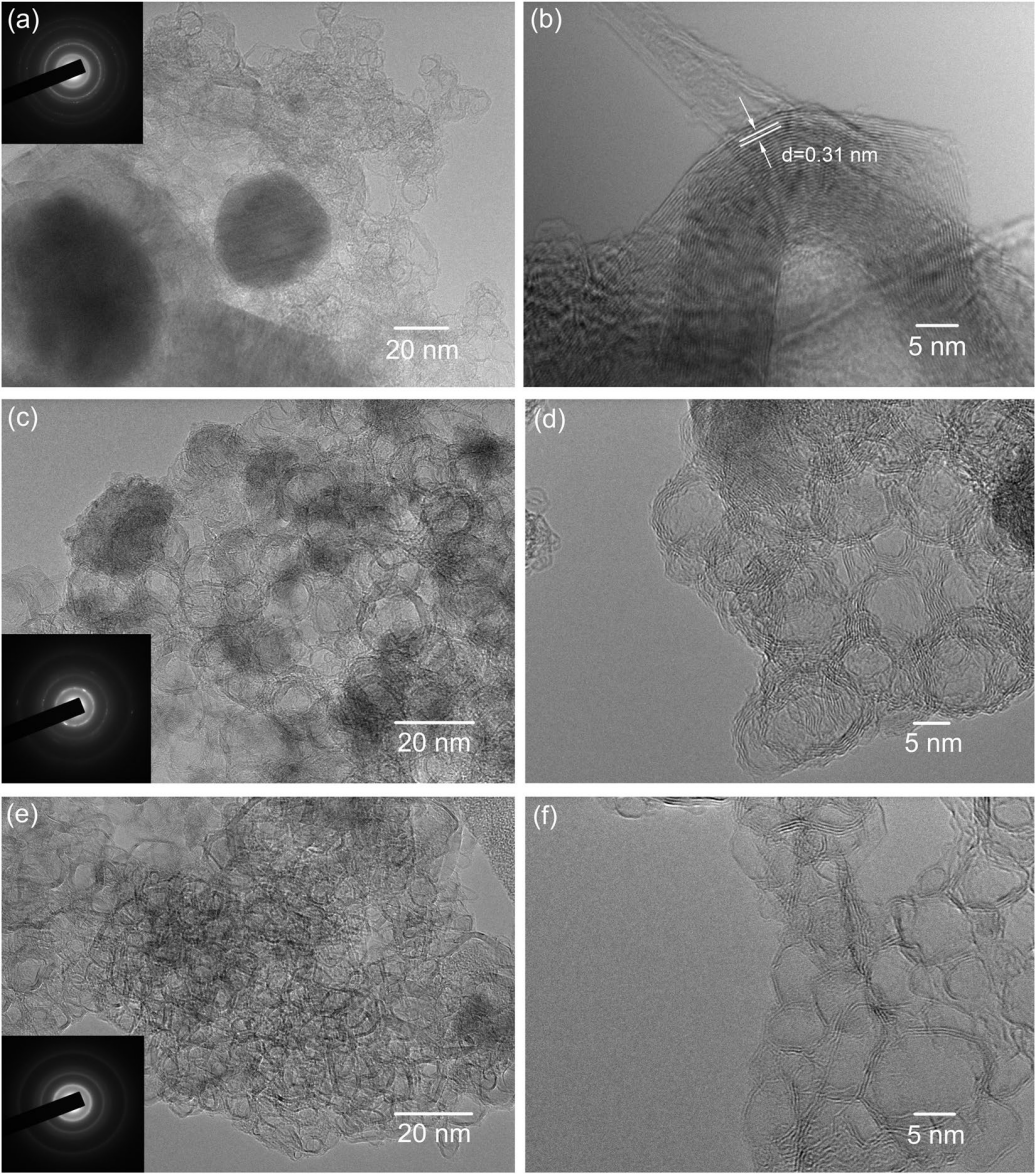
TEM images of the samples prepared under relatively high carbonization temperature. (**a,b**) Co@C-1200; (**c,d**) Co@C-1600 and (**e,f**) Co@C-2000. Inset: their SAED patterns.

**Figure 3:**
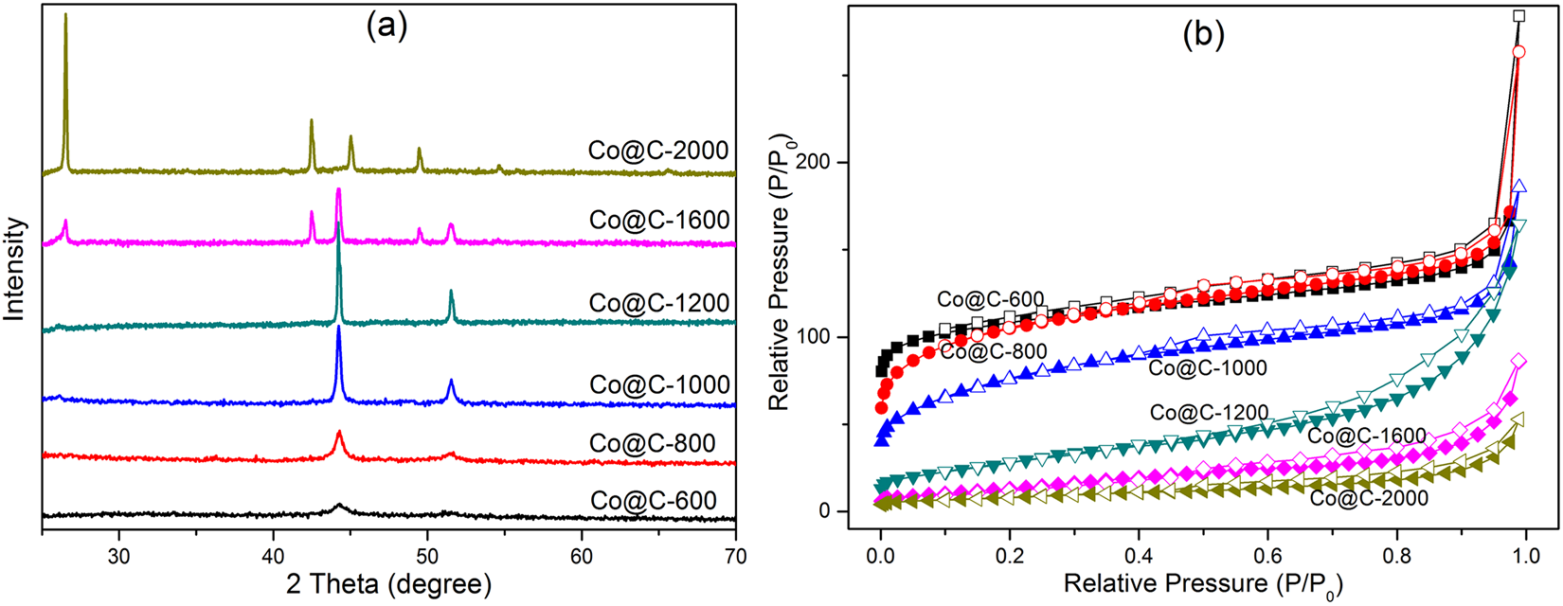
(**a**) XRD patterns, and (**b**) Nitrogen adsorption-desorption isotherm curves of the as-synthesized Co@C composites.

The precursor ZIF-67 has a particle size of approximately 400 nm with sodalite topology (Figure S1a)^39^. The structure and morphology of the Co@C composites were shown in Figs 1 and 2. After carbonization at 600 and 800 °C, samples Co@C-600 and Co@C-800 inherited the original morphology of ZIF-67, exhibiting a uniform particle size of 300 nm with a rough surface (Fig. 1a and c, and Figure S2). Sample Co@C-800 appeared to maintain the ZIF-67 backbone structure with slight deformation. However, the particles deformed further (shown in Fig. 1e) when the temperature increased up to 1000 °C. In sample Co@C-600, the darker dots of ~10 nm were dispersed well into the carbon matrix, which were Co nanoparticles according to selected area electron diffraction (SAED) (Fig. 1a,c, and e) and XRD (Fig. 3a) analyses. The Co nanoparticles tended to agglomerate to form larger particles at increased carbonization temperatures (800 °C and 1000 °C), resulting in improved crystallization of both Co and carbon and enhanced intensities in the SAED patterns (Fig. 1a,c, and e). Interestingly, carbon nanotubes on the surface of carbon matrix were observed during TEM investigation (Fig. 1b,d, and f), which were formed as a result of the Co catalytic effect during the carbonization^43^. The *in-situ* formation of carbon nanotubes could enhance the electronic conductivity of the composites^44^, being beneficial to the electrocatalytic performances. At low synthesis temperatures, the morphology of the resulting Co@C composites was generally in consistent with previous studies^26–28^.

Unlike the sample Co@C-600, Co@C-800 and Co@C-1000, the morphology of the Co@C composites generated at higher pyrolysis temperatures (≥1200 °C) was completely different (Figure S3). The ZIF-67 backbone was totally disappeared from samples Co@C-1200, Co@C-1600 and Co@C-2000. As shown in Fig. 2a for sample Co@C-1200, the Co nanoparticles of ~50 nm were wrapped with a few layers of graphite sheet, which was similar to Klose’s report, with a d-spacing of 0.31 nm corresponding to the distance of individual basal planes in graphite^38^. Samples Co@C-1600 and Co@C-2000 predominantly consisted of hollow carbon nano-onions of ~5–10 nm in sizes (Fig. 2c and e), which were *in-situ* generated under the catalytic effect of Co particles at high temperatures. The carbon nano-onion structures became more dominant and clearer for sample Co@C-2000 because of the higher crystallization degree. To the best of our knowledge, such small hollow carbon nano-onion structures have not been observed by using MOF as a precursor. Based on these TEM results, it is evident that the structures of MOF derived materials varied from Co-embedded in N-doped porous carbons/carbon nanotubes to hollow carbon nano-onions, depending on the carbonization temperatures.

The XRD patterns of the ZIF-67 precursor showed the pure sodalite crystallinity without any by-products (Figure S1b)^39^. After the thermal decomposition, the characteristic peaks of ZIF-67 cannot be observed from all Co@C samples, as shown in Fig. 3a. However, two peaks at 2θ = 44.2° and 51.6° for samples Co@C-600, Co@C-800, Co@C-1000, and Co@C-1200, indicated the presence of metallic Co (ICDD PDF #15–0806). These two peaks became stronger and sharper with the increase of carbonization temperatures, indicating the improved crystallinity and increases in particle sizes of Co^24^. The broad peak at around 2θ = 26° verified the existence of graphitic carbon. Sample Co@C-1600 exhibited not only the characteristic peaks of Co, but also three peaks at 2θ = 26°, 43° and 49.5°. The former two peaks were assigned to highly crystallized graphite carbon, and the latter small peak was indicative of the formation of cobalt carbide (ICDD PDF #89–2866)^45, 46^. Sample Co@C-2000 showed no peaks of Co, but exhibited the characteristic peaks of graphitic carbon at 2θ of 26°, 43° and 52°, and typical peaks at 2θ of 45.0 ° and 49.5 ° of cobalt carbide. These results confirmed that under high temperatures (≥1600 °C) metallic Co reacted with carbon and resulted in the formation of cobalt carbide.

The textural properties of the as-synthesized Co@C composites were assessed by the nitrogen sorption experiments (shown in Fig. 3b). The precursor ZIF-67 exhibited a typical type-I isotherm (Figure S1c), corresponding to the dominance of microporous structure. Samples Co@C-600, Co@C-800 and Co@C-1000 displayed similar isotherms to that of ZIF-67, and their higher N_2_ uptake at low relative pressure indicated the dominance of micropore. As a result, the relatively higher surface areas could provide better exposure and enhanced utilization of electroactive sites for the composite to exhibit higher elctrocatalytic activity. Further heat treatment at temperatures ≥1200 °C resulted in the pore collapse in the template and thus dramatically decreased BET surface area value. The carbonized samples Co@C-600, Co@C-800, Co@C-1000, Co@C-1200, Co@C-1600 and Co@C-2000 exhibited a specific surface area of 372, 369, 275, 102, 50 and 30 m^2 ^g^−1^, respectively. The reduced specific surface area and porosity of the composites at high carbonization temperature was due to the improved crystallinity. The slim hysteresis loops between the adsorption and desorption branches observed in all samples confirmed the existence of mesoporous feature originating from voids between particles.

**Figure 4:**
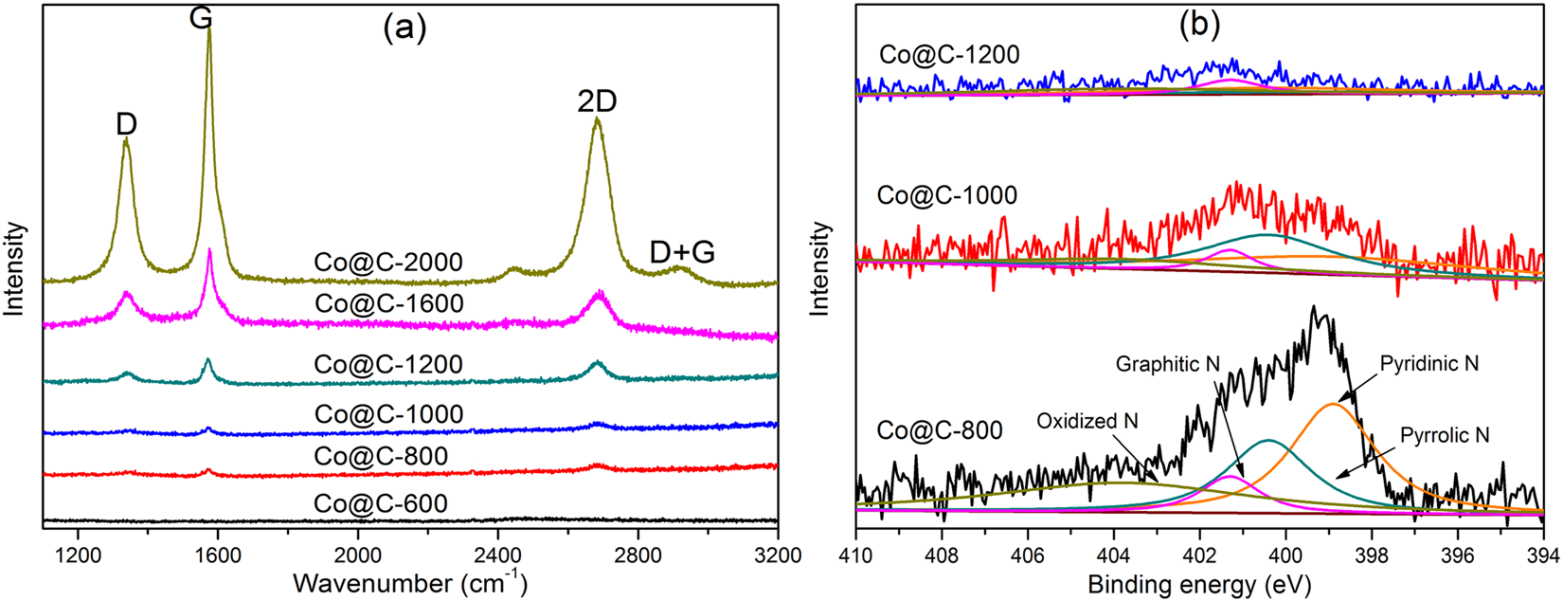
(**a**) Raman spectra, and (**b**) high-resolution N 1 s XPS spectra of the as-synthesized Co@C composites.

The Raman spectra (shown in Fig. 4a) of the as-synthesized Co@C composites exhibited the characteristic D and G bands at 1335 and 1590 cm^−1^, in which the D band was originated from the disordered carbon and the G band was associated with the graphic carbon, respectively^47^. The intensity ratio of the D and G band (I_D_/I_G_) of the samples decreased with the increase in carbonization temperatures, changing from 0.69, 0.60, 0.55, 0.46, 0.42 and 0.40 for samples Co@C-600, Co@C-800, Co@C-1000, Co@C-1200, Co@C-1600 and Co@C-2000 respectively. This is in line with that the applied carbonization temperature determined the graphitization degree, and high temperatures led to better graphitization in the Co@C composites^48^. Furthermore, the presence of distinct peaks at 2680 cm^−1^ (2D band) and 2900 cm^−1^ (D + G band) in the Raman spectrum of sample Co@C-2000 further confirmed the presence of highly graphitized carbon, i.e. the carbon nano-onions.

To probe the chemical compositions and their status in the as-synthesized Co@C composites, XPS measurement was then investigated. Element survey demonstrated the presence of C, N, O, and Co in all composites (Figure S4). Particularly, as an n-type dopant, nitrogen can facilitate the ORR and OER performances^49^. The high-resolution N 1 s spectrum in Fig. 4b can be deconvoluted into four characteristic peaks centred at binding energy of 398.9, 400.4, 401.3, and 403.8 eV, corresponding to pyridinic, pyrrolic, graphitic and oxidized nitrogen, respectively^50^. It is found that pyridinic N was dominated in sample Co@C-800 with a few pyrrolic N, less graphitic and oxidized N. The predominance of pyridinic and pyrrolic N could support the presence of Co-N_x_^24, 28^. Moreover, the pyrrolic/pyridinic N ratio increases in samples prepared at 1000 °C, which was in consistent with previous study^28^. The N content decreased dramatically when the carbonization temperature increased, possibly due to the high volatility of N species at high temperatures. The overall N content in sample Co@C-1200 was quite low, and all samples synthesized at relatively high temperatures (≥1200 °C) possessed negligible N species.

### Electrochemical ORR and OER evaluations

**Figure 5:**
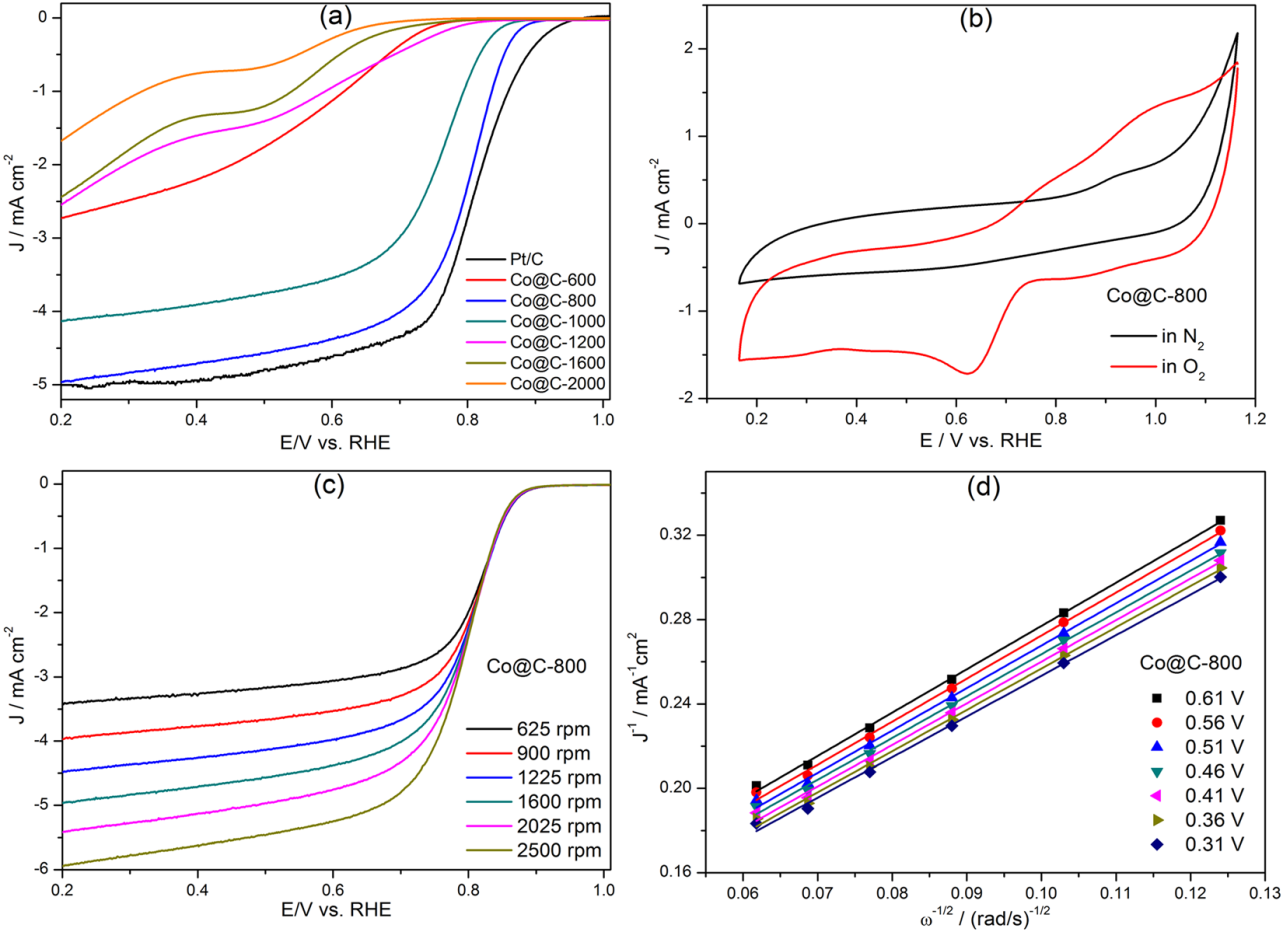
(**a**) LSV curves of the as-synthesized Co@C composites and Pt/C at 1600 rpm. (**b**) CV curves of Co@C-800. (**c**) Polarization curves of Co@C-800 measured at different rotation speeds. (**d**) K-L plots of Co@C-800 at different potentials.

The oxygen reduction properties of the as-synthesized Co@C composites were evaluated via electrochemical measurements of linear sweep voltammetry (LSV) curves. Sample Co@C-800 (Fig. 5a) exhibited the highest onset potential at 0.92 V and halfwave potential of 0.82 V, which were very close to the performance of the benchmark 20% Pt/C catalyst. The saturated current density of 5 mA cm^−2^ of sample Co@C-800 was also comparable to that of Pt/C. The ORR activity of sample Co@C-600 was lower than that of sample Co@C-800, probably due to the incomplete carbonization. It is worth noting that sample Co@C-800 exhibited the best electrocatalytic performance in ORR amongst other composites. Its excellent ORR performance is probably because of its large surface area, high N content and features with Co particles embedded in N-doped porous carbons/carbon nanotubes that may benefit to the formation of CoN_x_ species^19, 22, 23^. Samples generated at even higher carbonization temperatures >800 °C, possess more highly graphitized carbon species, accompanied with severer Co nanoparticle aggregation and less N species. Although, on one side, proper graphitization of carbon species could increase the electroconductivity and improve the ORR activity; on the other side, excessive graphitization would damage the porous structure and decrease the surface area as confirmed by the gas sorption analysis, and the Co particles aggregation as well as the volatilization of N species, were all detrimental to the ORR performance.

The electrocatalytic activities of sample Co@C-800 were further evaluated using cyclic voltammetry (CV) measurements at 25 °C in N_2_- and O_2_-saturated 0.1 M KOH electrolyte solution. No obvious redox peak was observed in the N_2_-saturated solution (Fig. 5b); whilst a well-defined cathodic peak appeared at 0.64 V in the O_2_-saturated solution, indicating the ORR active feature. Moreover, the ORR performances of sample Co@C-800 were also evaluated using a rotating disk electrode (RDE) under rotation speeds varying from 625 to 2500 rpm. An increase in the rotation speed resulted in higher current densities (Fig. 5c), owing to the improved diffusion for oxygen to reach the electrode surface. Based on RDE results under different rotating speeds, Koutecky–Levich (K–L) plots were presented in Fig. 5d. It is clear that sample Co@C-800 exhibited a good linearity and near parallelism characteristics in the K–L plots (shown in Fig. 5d). According to K–L equation, the electron transfer number for sample Co@C-800 was close to 4.0, suggesting that the ORR proceeded through a four-electron pathway.

**Figure 6:**
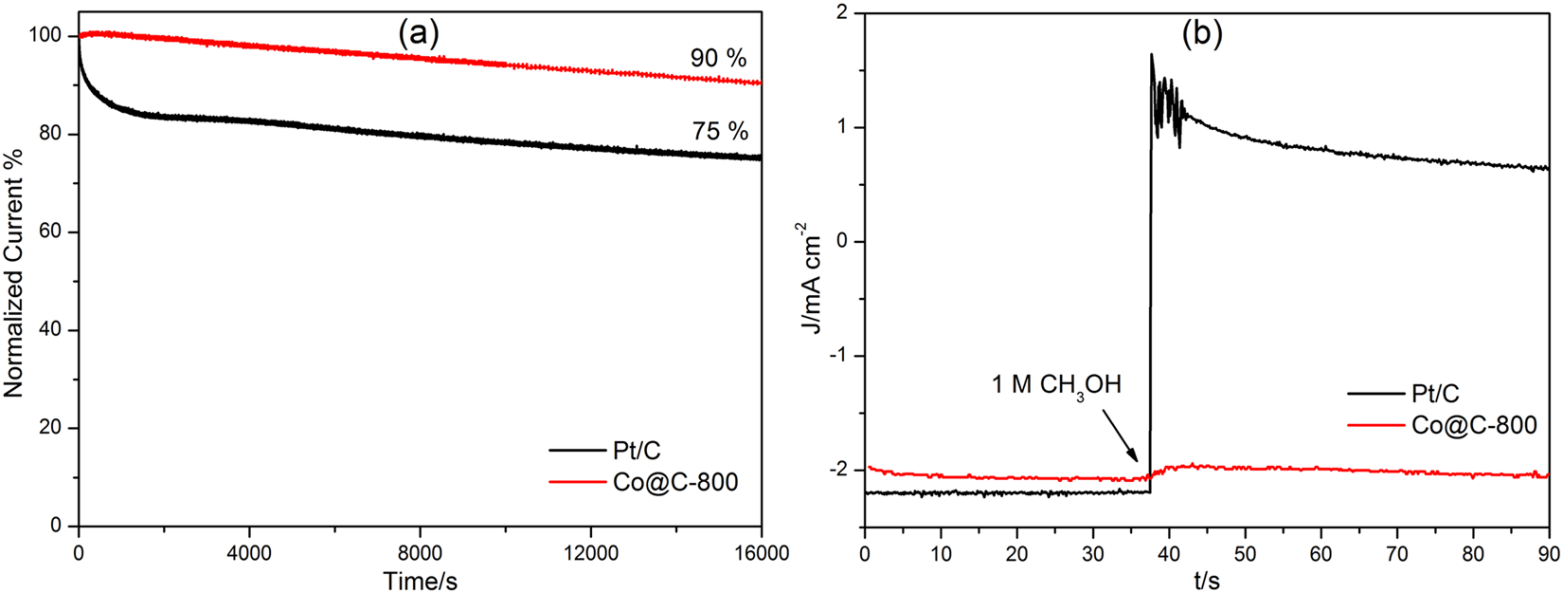
(**a**) Chronoamperometric responses at a 0.8 V constant potential, (**b**) Chronoamperometric responses at 0.8 V followed by the addition of methanol.

The stabilities of the Co@C-800 composite and Pt/C in ORR were evaluated at 0.8 V in 0.1 M KOH solution. After running for 16000 s, sample Co@C-800 still remained 90% its initial current density, whilst the Pt/C dropped to 75%, as shown in Fig. 6a, clearly being outperformed. In addition, 1 M methanol was added into a 0.1 M KOH electrolyte solution to investigate the methanol tolerance of sample Co@C-800 and Pt/C (Fig. 6b), and the results showed that Pt/C catalyst was again outperformed with a sharp decrease in current density, while only a negligible effect was observed for sample Co@C-800. The high ORR stability and excellent methanol tolerance of Co@C-800 may be attributed to the protection role of the carbon matrix.

**Figure 7:**
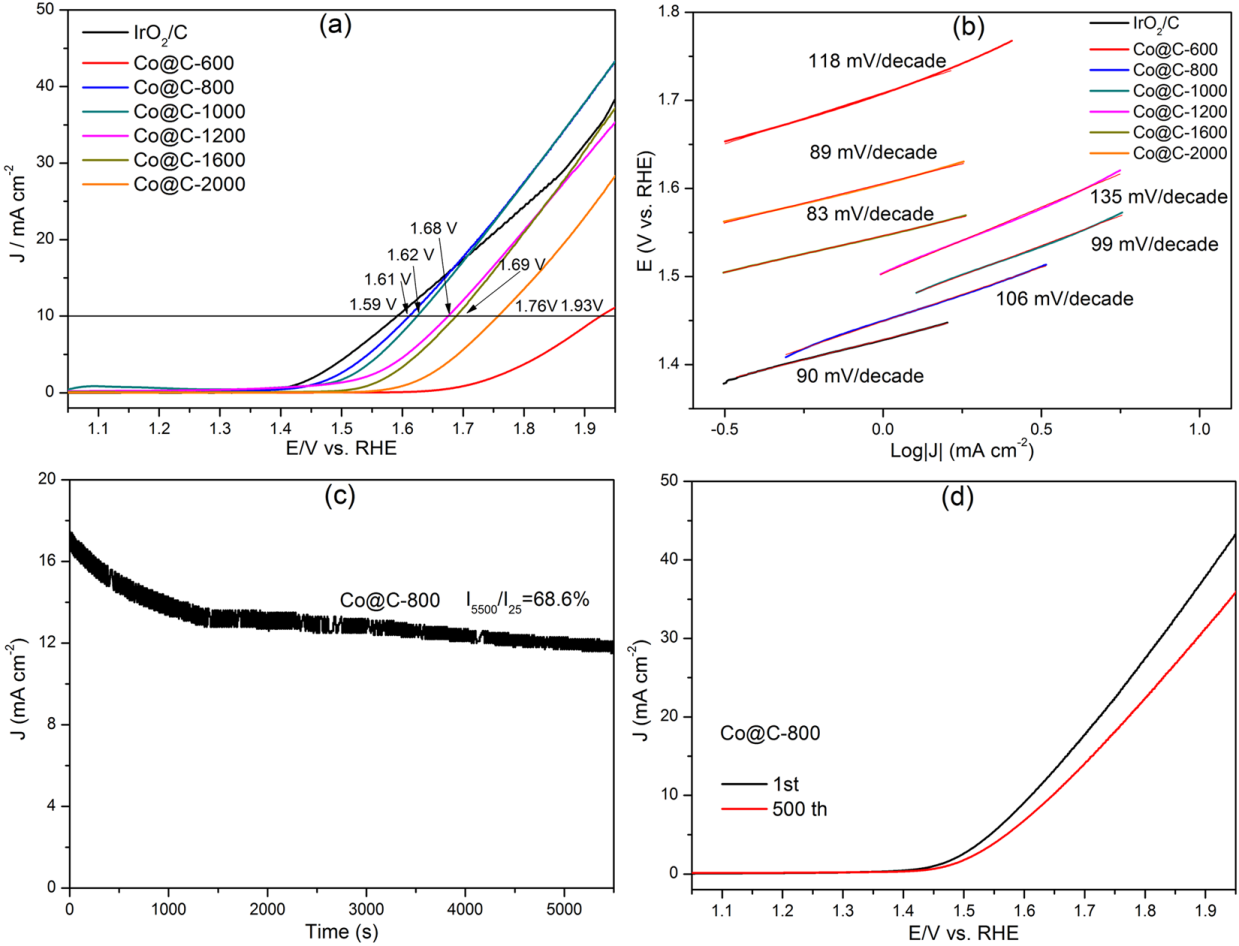
(**a**) LSV curves and (**b**) the corresponding Tafel plots of the as-synthesized Co@C composites and IrO_2_/C at 5 mV s^−1^. (**c**) Chronoamperometric response for Co@C-800 at 1.7 V. (**d**) Polarization curves of Co@C-800 before and after 500 scan cycles.

The electrocatalytic performances of Co@C composites towards oxygen evolution reaction (OER) were also evaluated in 0.1 M KOH solution and their linear sweep voltammetry were presented in Fig. 7a. Sample Co@C-800 demonstrated a small onset potential at 1.43 V, which was only marginally higher than that of IrO_2_/C catalyst (1.42 V). Notably, the current density of IrO_2_/C even dropped below that of Co@C-800 at potentials higher than 1.68 V, indicating a highly catalytic active towards OER of sample Co@C-800. Indeed, sample Co@C-800 exhibited the highest OER current density and the earliest onset of catalytic current amongst all the Co@C samples studied. As shown in Fig. 7a, samples Co@C-600, Co@C-800, Co@C-1000, Co@C-1200, Co@C-1600 and Co@C-2000 required a potential of 1.93, 1.61, 1.62, 1.68, 1.69 and 1.76 V, respectively, to achieve 10.0 mA cm^−2^. The value of sample Co@C-800 (1.61 V) is comparable to that for IrO_2_/C (1.59 V), lower than that of other reported catalysts, including N-doped graphene/cobalt/carbon hybrid (1.66 V)^25^, N-doped graphene/carbon nanotube composite (>1.65 V)^51^, Mn_3_O_4_/CoSe_2_ hybrid (1.68 V)^52^, and electrochemical post-treatment of the metallic cobalt nanospheres coated with an ultrathin carbon layer (1.56 V)^53^.

The linear fittings of the Co@C composites and IrO_2_/C were calculated to analyse their kinetics for OER (Fig. 7b). Generally, Co@C composites exhibited relatively low Tafel slopes ranging from 83 to 135 mV dec^−1^, implying good OER kinetics. The carbonization temperatures had a significant effect on the OER activities of the samples. For sample Co@C-800, the presence of Co and N-dopants could render the adjacent carbon atoms positively charged, facilitating adsorption of OH^−^ and promoting the electron transfer^54, 55^. Its high surface area could also benefit to the maximum exposure of electroactive sites. However, the Tafel slope value in this paper are still higher than the reported value (50–70 mV/decade) by most literatures. It is supposed that Co hydroxide or oxyhydroxide would be formed during OER in KOH electrolyte, which might be the key reason for the high Tafel slope.

The stability of Co@C-800 was then assessed by the chronoamperometric test. Sample Co@C-800 remained 68.6% its initial current density after 5500 s at 1.7 V (Fig. 7c). In addition, in a further stability test, Co@C-800 shows only a small drop of the current density after 500 potential cycles (Fig. 7d), indicating a good durability towards OER. During the OER, metallic Co would be partially oxidized into cobalt oxide to form cobalt oxide/complex species, which could decay the OER activity^56, 57^. However, in the Co@C-800 composite, the Co nanoparticles were homogeneously embedded within the carbon matrix, being protected by surrounding carbon therefore retarding the oxidation.

## Conclusion

In summary, a series of composites of cobalt-embedded in N-doped porous carbons, carbon nanotubes or hollow carbon nano-onions via one-step carbonization of ZIF-67 have been successfully prepared. The effect of carbonization temperatures on the morphologies and electrocatalytic properties of resultant products is systematically studied in this work. Under lower carbonization temperatures (600–1000 °C), cobalt-embedded in N-doped porous carbons or carbon nanotubes composites were formed, accompanied with the maintaining of high surface area of porous carbons, N species and small cobalt nanoparticles. Consequently, enhanced electrocatalytic performances can be achieved. While cobalt-embedded in hollow graphitic carbon onions composites with low surface area, negligible nitrogen content, aggregated cobalt nanoparticles and low electrocatalytic performances were generated under higher carbonization temperature (1200–2000 °C). Due to the hierarchical porous carbon structure, N-doping effect and the homogeneous cobalt dispersion, sample Co@C-800 exhibits the best electrocatalytic activities towards both ORR and OER with good stability amongst the resultant nanocomposites. Our findings may pave a new way to design novel porous carbon-based non-precious metal as bifunctional ORR and OER electrocatalysts for the next generation energy storage and conversion devices.

## Electronic supplementary material

Supplementary Information
