# Supplementary material for: Metal-organic-frameworks derived cobalt embedded in various carbon structures as bifunctional electrocatalysts for oxygen reduction and evolution reactions
Source: Sci Rep. 2017 Jul 13;7:5266. doi: 10.1038/s41598-017-05636-y (PMC5509653; doi:10.1038/s41598-017-05636-y)
Supplement: Supplementary file 1 — Supplementary Information [file 41598_2017_5636_MOESM1_ESM.pdf]

Supplementary information

Metal-organic-frameworks derived cobalt embedded in various carbon structures as bifunctional electrocatalysts for oxygen reduction and evolution reactions

Binling Chen[1], Guiping Ma[1,2], Yanqiu Zhu[1] & Yongde Xia[1,*]

[1] *College of Engineering, Mathematics and Physical Sciences, University of Exeter, Exeter EX4 4QF, United Kingdom*

[2] *State Key Laboratory of Chemical Resource Engineering, Beijing Laboratory of Biomedical Materials, Beijing University of Chemical Technology, Beijing 100029, P R China*

*Address correspondence to Yongde Xia, y.xia@exeter.ac.uk

Koutecky-Levich plots were analysed at different potentials. The number of electrons transferred (n) can be obtained from the slopes of their linear-fit lines based on the K-L equations.

$$\frac{1}{J} = \frac{1}{J_{\mathrm{K}}} + \frac{1}{B\omega^{1/2}}$$

$$B = 0.62nFC_0D_0^{2/3}\upsilon^{-1/6}$$

Where J is the measured current density, J_{K} is the kinetic current density, B is the Levich constant, ω is the angular velocity of the rotating electrode, n is the overall number of electron transferred in the ORR process, F is the Faraday constant (96485 C mol^{-1}), C_0 is the bulk concentration of O_2 (1.2×10^{-6} mol cm^{-3}), D_0 is the diffusion coefficient of O_2 (1.9×10^{-5} cm^2 s^{-1}) and υ is the kinematic viscosity (0.01 cm^2 s^{-1}) of the electrolyte.

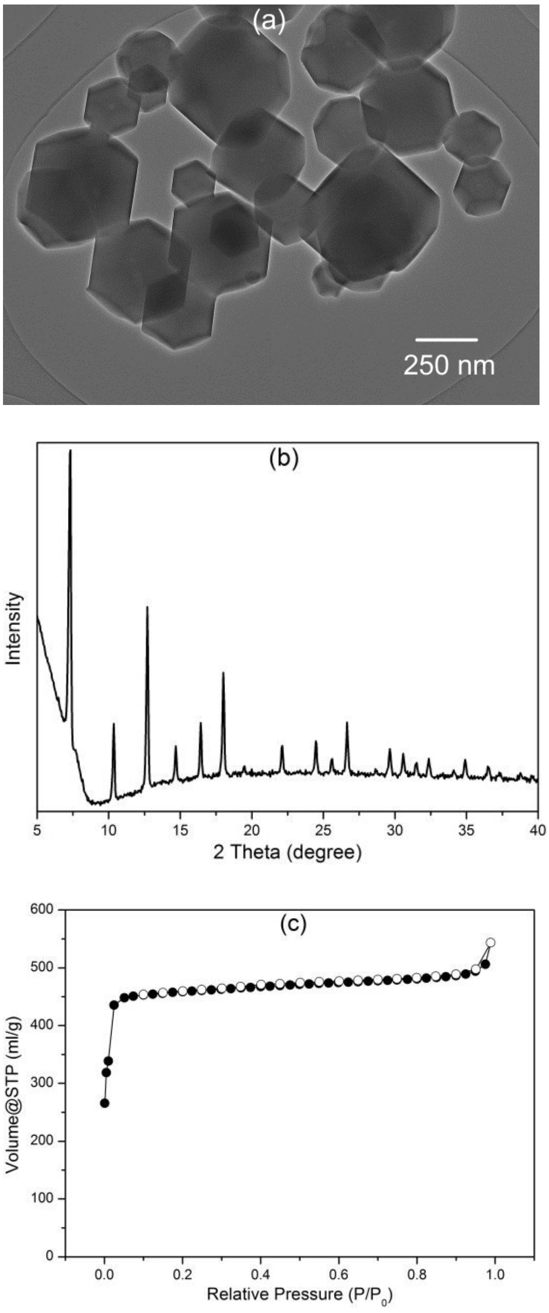

Figure S1. (a) TEM image, (b) powder XRD pattern and (c) nitrogen sorption isotherms of the parental ZIF-67 material.

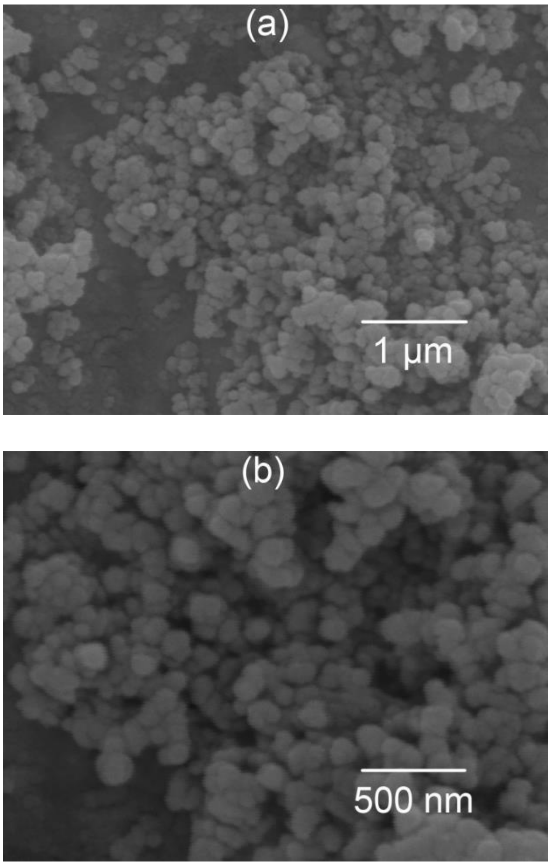

Figure S2. SEM images of the as-synthesized Co@C-800 nanocomposite under (a) low and (b) high magnification.

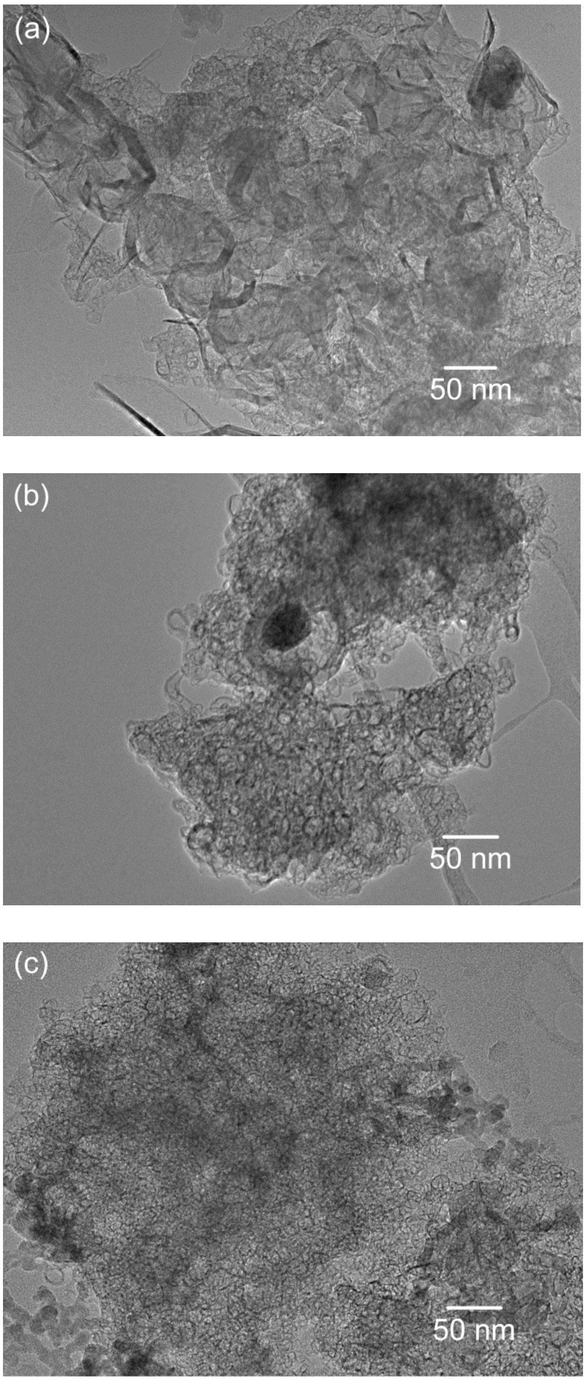

Figure S3. Low-magnification TEM images of the samples generated under relatively low carbonization temperatures: (a) Co@C-1200; (b) Co@C-1600 and (c) Co@C-2000.

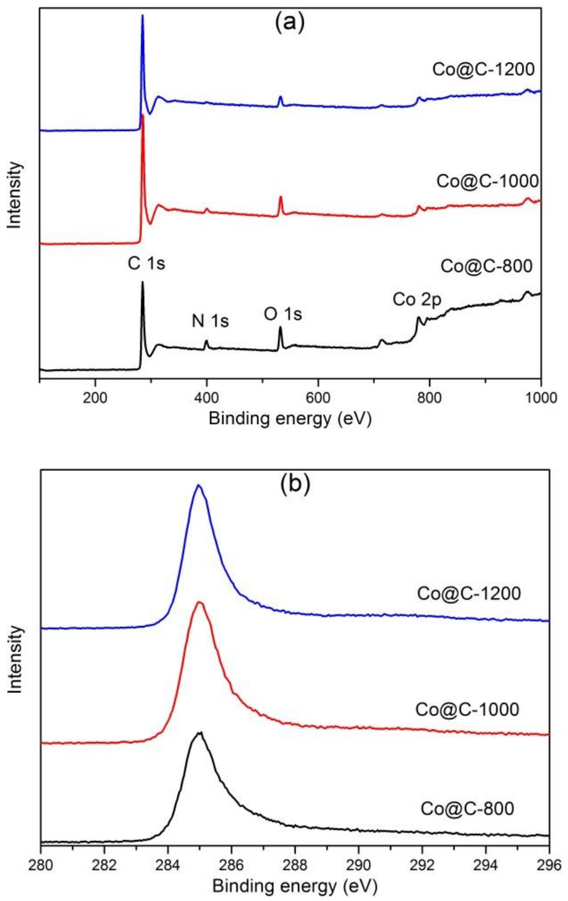

Figure S4. (a) Element surveys by XPS and (b) high-resolution XPS spectrum of C 1s for the as-synthesized nanocomposites.
